# Phosphorylation of VP1 Mediated by CDK1-Cyclin B1 Facilitates Infectious Bursal Disease Virus Replication

**DOI:** 10.1128/jvi.01941-22

**Published:** 2023-01-05

**Authors:** Xifeng Hu, Zheng Chen, Xiangdong Wu, Zhen Ding, Yu Huang, Qiuling Fu, Zhen Chen, Huansheng Wu

**Affiliations:** a Department of Veterinary Preventive Medicine, College of Animal Science and Technology, Jiangxi Agricultural University, Nanchang, People’s Republic of China; b Jiangxi Provincial Key Laboratory for Animal Health, College of Animal Science and Technology, Jiangxi Agricultural University, Nanchang, People’s Republic of China; c Institute of Animal Husbandry and Veterinary Medicine of Fujian Academy of Agricultural Sciences, Fuzhou, People’s Republic of China; Instituto de Biotecnologia/UNAM

**Keywords:** IBDV VP1, CDK1-cyclin B1, phosphorylation, Ser7, polymerase activity, viral polymerase, host kinase, viral protein phosphorylation, virology, virus replication

## Abstract

Infectious bursal disease virus (IBDV) is a double-stranded RNA (dsRNA) virus belonging to the genus *Avibirnavirus* in the family *Birnaviridae*. It can cause serious failure of vaccination in young poultry birds with impaired immune systems. Post-translational modifications of the VP1 protein are essential for viral RNA transcription, genome replication, and viral multiplication. Little information is available so far regarding the exact mechanism of phosphorylation of IBDV VP1 and its significance in the viral life cycle. Here, we provide several lines of evidence that the cyclin-dependent kinase 1 (CDK1)-cyclin B1 complex phosphorylates VP1, which facilitates viral replication. We show that the CDK1-cyclin B1 specifically interacts with VP1 and phosphorylates VP1 on the serine 7 residue, located in the N-terminal ^7^SPAQ^10^ region, which follows the optimal phosphorylation motif of CDK1, p-S/T-P. Additionally, IBDV infection drives the cytoplasmic accumulation of CDK1-cyclin B1, which co-localizes with VP1, supporting the kinase activity of CDK1-cyclin B1. Treatment with CDK1 inhibitor RO3306 and knockdown of CDK1-cyclin B1 severely disrupts the polymerase activity of VP1, resulting in diminished viral replication. Moreover, the replication of S7A mutant recombinant IBDV was significantly decreased compared to that of wild-type (WT) IBDV. Thus, CDK1-cyclin B1 is a crucial enzyme which phosphorylates IBDV VP1 on serine 7, which is necessary both for the polymerase activity of VP1 and for viral replication.

**IMPORTANCE** Infectious bursal disease virus still poses a great economic threat to the global poultry farming industry. Detailed information on the steps of viral genome replication is essential for the development of antiviral therapeutics. Phosphorylation is a common post-translational modification in several viral proteins. There is a lack of information regarding the significance of VP1 phosphorylation and its role in modulating the viral life cycle. In this study, we found that CDK1-cyclin B1 accumulates in the cytoplasm and phosphorylates VP1 on serine 7. The presence of a CDK1 inhibitor and the silencing of CDK1-cyclin B1 decrease IBDV replication. The mutation of VP1 serine 7 to alanine reduces VP1 polymerase activity, disrupting the viral life cycle, which suggests that this residue serves an essential function. Our study offers novel insights into the regulatory mechanism of VP1 phosphorylation.

## INTRODUCTION

**FIG 1:**
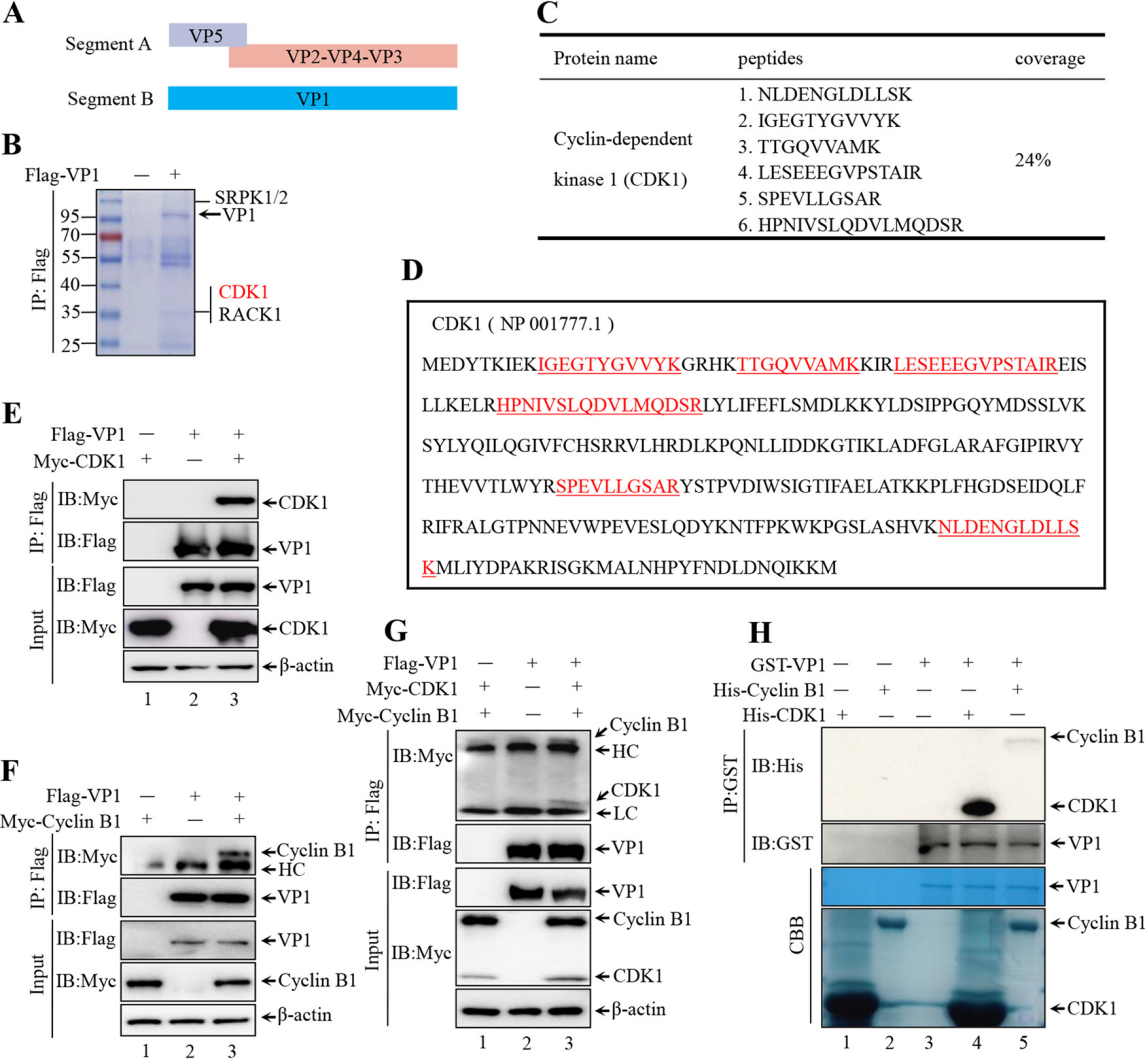
Cyclin-dependent kinase 1 (CDK1)-cyclin B1 complex interacts with VP1 (A) Schematic diagram of the infectious bursal disease virus (IBDV) genome. (B) HEK-293T (293T) cells transiently transfected with Flag-VP1 (empty vectors as a negative control). Flag-VP1 was immunoprecipitated (IP) with anti-Flag agarose, separated by SDS-PAGE and stained with Coomassie brilliant blue. Separated specific bands were excised and analyzed by mass spectrometry. Four host kinases were associated with VP1, including cyclin-dependent kinase 1. (C) Coverage of identified peptides in the complete length of CDK1. (D) Identified peptides located in the CDK1 sequence. (E) Immunoblotting (IB) of whole-cell lysates (bottom) and proteins immunoprecipitated with anti-Flag agarose (top) from 293T cells transfected with the indicated plasmids for 36 h. (F) 293T cells were co-transfected with Flag-VP1- and Myc-cyclin B1-expressing plasmids for 36 h. Cells were then lysed and IP by anti-Flag agarose. The IP complex and whole-cell lysates were subjected to Western blotting using the indicated antibodies. (G) IB of whole-cell lysates (bottom) and proteins immunoprecipitated with anti-Flag agarose (top) from 293T cells co-transfected with Flag-VP1-, Myc-CDK1-, and Myc-cyclin B1-expressing plasmids for 36 h. (H) Purified GST-VP1 protein was separately incubated with purified His-CDK1 and His-cyclin B1 protein, followed by IP with glutathione *S*-transferase (GST) resin. IP complex was analyzed by Western blotting with indicated antibodies. The whole protein was examined by SDS-PAGE and visualized by Coomassie brilliant blue staining.

Infectious bursal disease (IBD), originally identified as Gumboro disease, is an extremely contagious immunosuppressive disease in young chickens caused by infectious bursal disease virus (IBDV) (1). IBDV is the type species of the family *Birnaviridae*, which includes nonenveloped, icosahedral, double-stranded RNA (dsRNA) viruses that infect a variety of vertebrate and invertebrate hosts (2). The IBDV genome consists of two dsRNA segments, A and B (Fig. 1A) (3). Segment A, about 3.2 kb in length, has two partially overlapping open reading frames (ORFs) (4). The smaller ORF encodes the nonstructural protein VP5, which is dispensable for viral replication (5). The larger ORF encodes a polyprotein, pVP2-VP4-VP3, which is autocatalytically processed by VP4 to produce VP2, VP3, and VP4 (6, 7). VP3 is a structural protein that interacts with both VP1 and viral genomic dsRNA to form a viral replication complex (8). Segment B, about 2.8 kb, encodes VP1, also called RNA-dependent RNA polymerase (9, 10).

The phosphorylation of viral proteins plays an essential role in regulating their functions and the viral life cycle (11). Viral polymerase core protein PB1 is phosphorylated by protein kinase C, which is required for replication in avian influenza virus (12, 13). A critical phosphorylation site in the polymerase of *Turnip yellow mosaic virus* (14), dengue virus 2 (15), and *Cucumber mosaic virus* was found to regulate viral genomic transcription (16). Phosphorylation of the Ser156 residue of respiratory syncytial virus phosphoprotein facilitates viral genomic replication (17). Phosphorylation of the Marburg virus VP30 protein suppresses viral genomic transcription (18). Similarly, Ebola virus VP30 regulates viral RNA replication via dynamic phosphorylation (19–21). Therefore, studies on the phosphorylation of critical residues of the IBDV polymerase protein which affect its enzymatic activity would help us to better understand viral genome and viral replication.

IBDV VP1 is a viral polymerase which has roles in transcription, viral genome replication, and mature virion assembly (22). There are two forms of VP1 in the mature virion: dsRNA-unconjugated VP1 (also called free VP1) and dsRNA-conjugated VP1 (also called VP1g) (23, 24). Following virus entry into the host cell, these two forms of VP1, along with VP3, are released into the cytoplasm, where they initiate transcription and viral genome replication (25). Previous reports have shown that VP1 undergoes ubiquitination, SUMOylation, and self-guanylylation to perform various functions (26–28). However, the role of phosphorylation of VP1 in regulating its polymerase activity and the viral life cycle is still unclear.

The activity of cyclin-dependent kinase (CDK)-cyclin complex is essential for the cell cycle (29). The kinase activity of CDK1-cyclin B1 is controlled by the phosphorylation and dephosphorylation of CDK1 at T161 and Y15, respectively (30, 31). Phosphorylation/dephosphorylation of Y15 is catalyzed by the kinases of the Wee1/Myt1 family and the phosphatases of the CDC25C family (32, 33). Apart from cell cycle regulation, CDK1-cyclin B1 is also known to be involved in regulating IFN-β production and viral genome replication (34). The human parvovirus B19 NS1 protein induces G_2_-phase arrest by inactivating cyclin B-CDK1 complex (35). The avian reovirus p17 protein inhibits CDK1-mediated vimentin phosphorylation, which causes cell cycle arrest and facilitates virus replication (36). CDK1 also regulates antiviral immunity against HIV-1 infection by maintaining the phosphorylation of the antiviral factor SAMHD1 (37). The CDK2-cyclin B1 complex supports hepatitis B virus replication by phosphorylating the viral core protein on its C-terminal domain (38). Whether or not CDK1-cyclin B1 is involved in regulating IBDV infection by phosphorylating viral proteins is still unclear.

In this study, we sought to investigate the role of CDK1-cyclin B1 in VP1 phosphorylation. IBDV infection induces CDK1-cyclin B1 complex accumulation in the cytoplasm. CDK1 inhibitor treatment and CDK1-cyclin B1 silencing were used to further confirm its role in IBDV replication. CDK1-cyclin B1 kinase complex specifically interacts and phosphorylates VP1. After identifying the phosphorylation site of VP1 on the serine 7 residue and its effect on polymerase activity, we rescued an S7A mutant in full-length viruses and further examined its role in viral growth kinetics.

## RESULTS

### CDK1-cyclin B1 interacts with VP1.

Previous studies have shown that the phosphorylation of several viral polymerases is critical for genome replication (39). To examine the role of VP1 phosphorylation, we first investigated possible phosphorylation kinases interacting with VP1. Purified Flag-VP1 from transfected HEK-293T cells (here, 293T cells) (empty plasmid as a negative control) was subjected to mass spectrometry analysis. As shown in Fig. 1B, four host kinases were associated with VP1, including cyclin-dependent kinase 1 (CDK1), an essential kinase that regulates the cellular life cycle (40). Data from mass spectrometry analysis showed six peptides with 24% coverage of human CDK1 (Fig. 1C and D). In addition, the homology of CDK1 is highly similar, with 90.43% similarity between chickens and humans (data not shown). To further confirm the interaction between VP1 and CDK1, we performed a co-immunoprecipitation (co-IP) assay with 293T cells co-transfected with Flag-VP1 and Myc-CDK1, and immunoblotting of the IP complex confirmed the interaction between CDK1 and VP1 (Fig. 1E, lane 3). The CDK1 is the catalytic subunit of the highly conserved protein kinase complex CDK1-cyclin B1, which plays a critical role in regulating the cell cycle (41). Increasing evidence suggests that several host and viral proteins are common targets of CDK-cyclin B1 (42, 43). We next analyzed the interaction of VP1 and cyclin B1 using a co-IP assay (Fig. 1F, lane 3) to further confirm the association of VP1 with CDK1-cyclin B1 complex. Myc-CDK1 and Myc-cyclin B1 were co-transfected with Flag-VP1 into 293T cells for co-IP and Western blotting. We detected both CDK1 and cyclin B1 protein in a Western blot of the IP complex (Fig. 1G, lane 3), suggesting that VP1 could interact with CDK1-cyclin B1 complex. Glutathione *S*-transferase (GST)-pulldown assays with purified GST-VP1 and His-tagged CDK1-cyclin B1 confirmed the association of VP1 and CDK1-cyclin B1 complex *in vitro* (Fig. 1H, lanes 4 and 5). In conclusion, our data suggest that CDK1-cyclin B1 kinase complex interacts with IBDV VP1.

### IBDV infection drives cytoplasmic accumulation of active CDK1-cyclin B1.

**FIG 2:**
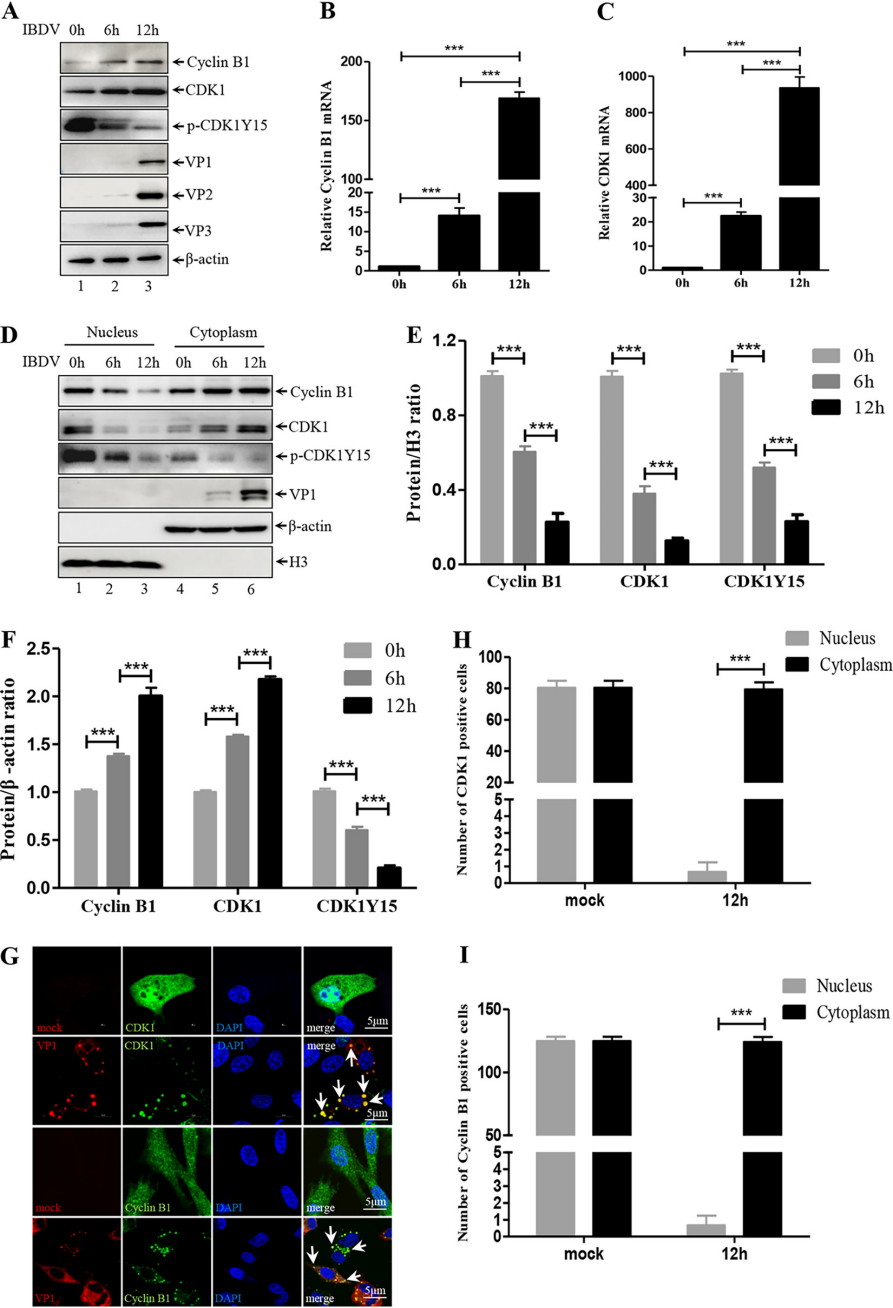
IBDV infection promotes expression of CDK1-cyclin B. (A) DF-1 cells were infected with IBDV at different times. Lysates of the infected DF-1 cells were subjected to Western blotting with the indicated antibodies. (B and C) Real-time quantitative PCR measurements of *CDK1* (B) and *cyclin B1* (C) mRNA abundances at the indicated times post-IBDV infection. Expression was normalized to the *gapdh* mRNA level. (D) DF-1 cells were infected with IBDV (MOI = 1) at the indicated times. Nuclear and cytoplasmic proteins were separated. CDK1, cyclin B1, CDK1Y15, VP1, β-actin and H3 protein level were analyzed by Western blotting. (E and F) Nuclear (E) and cytoplasmic (F) Western blot data were semi-quantified and normalized against β-actin and H3 protein loading control, respectively. (G) Confocal microscopy immunofluorescence images of mock-infected or IBDV (red)-infected DF-1 cells transfected with Myc-CDK1 (green) or Myc-cyclin B1 (green). Nuclei were stained with DAPI (4′,6-diamidino-2-phenylindole; blue). White arrows indicate VP1 puncta which co-localized with CDK1 or cyclin B1. Scale bars = 5 μm. (H and I) Quantified numbers of CDK1- (H) and cyclin B1-positive (I) cells in the nucleus and cytoplasm. Data were obtained from three independent experiments (*n* = 3) and are presented as means ± standard deviation (SD). ***, *P < *0.001.

To study the role of CDK1-cyclin B1 in the IBDV life cycle, we analyzed the RNA and protein levels of CDK1-cyclin B1 in DF-1 cells infected with IBDV. We found that protein and mRNA levels of CDK1-cyclin B1 were significantly increased after IBDV infection in a time-dependent manner (Fig. 2A to C). CDK1 activity is regulated by several kinases and phosphatases. De-phosphorylation of CDK1 at Tyr15 (Y15) by several phosphatases facilitates the kinase activity of CDK1 (44). Interestingly, the level of p-CDK1Y15 was significantly decreased during IBDV infection (Fig. 2A), suggesting that the kinase activity of CDK1 was significantly increased during IBDV infection. It has been previously determined that during viral infection, the subcellular distribution of CDK1-cyclin B1 is altered, which is essential for the active CDK1-cyclin B1 to regulate virus replication (45). Subcellular fractionation of protein from the nucleus and cytoplasm was performed to analyze the subcellular distribution of CDK1-cyclin B1 and p-CDK1Y15 during IBDV replication. We found that CDK1-cyclin B1 distributed in both the nucleus and cytoplasm during the resting state, whereas it accumulated in the cytoplasm after exposure to IBDV and almost disappeared in the nucleus after 12 h of IBDV infection (Fig. 2D to F). In addition, the level of p-CDK1Y15 in the cytoplasm was significantly reduced during IBDV infection, suggesting that IBDV infection facilitated CDK1 kinase activity in the cytoplasm (Fig. 2D to F). To further confirm the distribution of CDK1-cyclin B1, we analyzed the subcellular localization of CDK1-cyclin B1 after IBDV infection by confocal microscopy. Consistent with the immunoblotting data, CDK1 and cyclin B1 accumulated in the cytoplasm, co-localized with VP1 puncta, and disappeared in the nucleus after IBDV infection (Fig. 2G). Enumeration of CDK1-cyclin B1-positive cells showed that IBDV infection caused the accumulation of CDK1-cyclin B1 in the cytoplasm (Fig. 2H and I). These findings suggest that IBDV infection promotes the accumulation of CDK1-cyclin B1, which co-localizes with VP1 in the cytoplasm, and simultaneously facilitates CDK1 kinase activity, which may be essential for IBDV replication.

### CDK1 inhibitor impairs IBDV replication.

**FIG 3:**
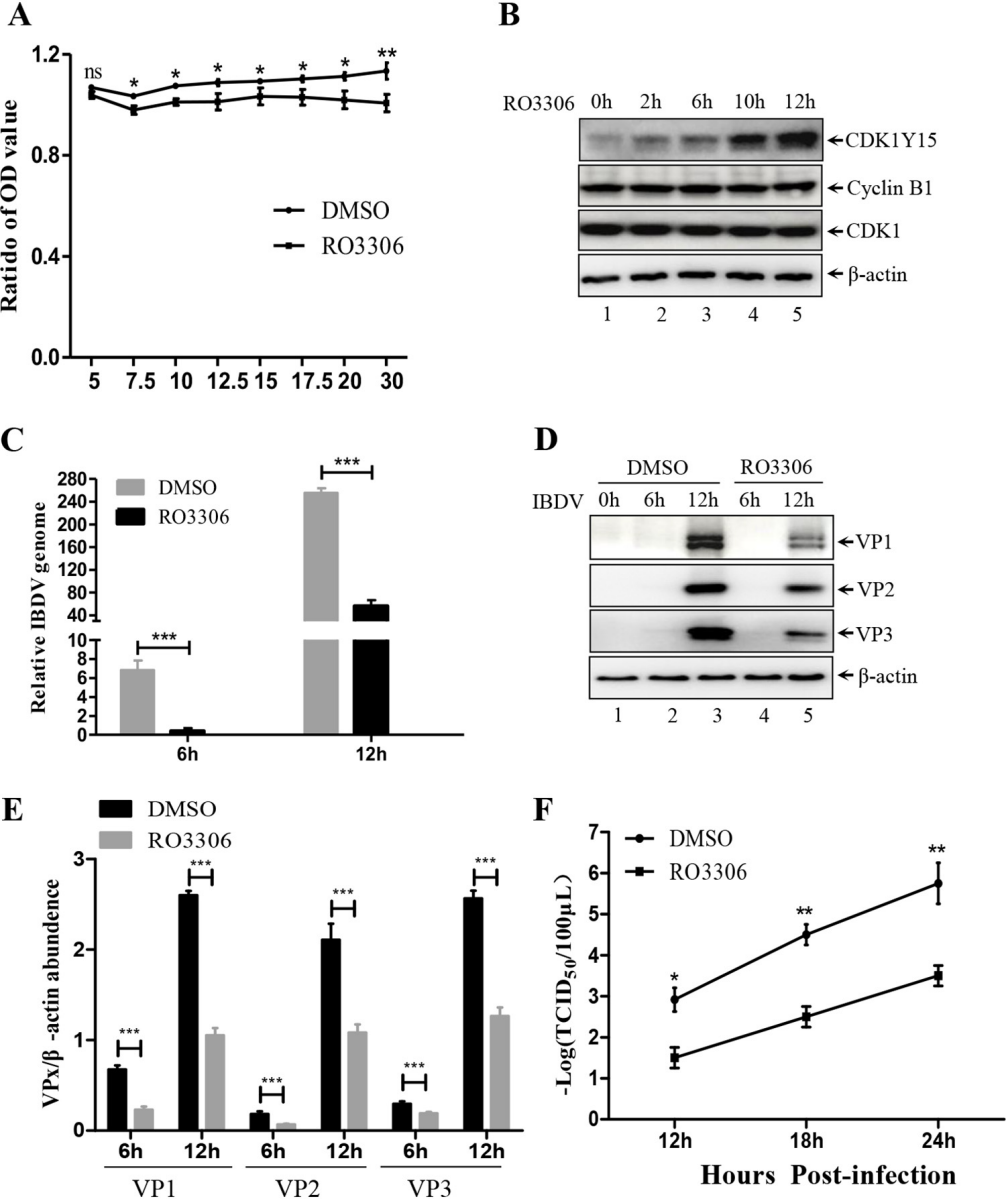
CDK1 inhibitor impairs IBDV replication. (A) DF-1 cells were treated with the indicated concentrations of RO3306 or dimethyl sulfoxide (DMSO). Treated cells were subjected to a cell viability assay as determined by a CCK8 kit. (B) DF-1 cells were treated with 5 μg/mL RO3306 or DMSO for 2, 6, 10, and 12 h. Whole-cell lysates were collected for Western blot analysis with the indicated antibodies. (C–D) DF-1 cells treated with 5 μg/mL RO3306 or DMSO for 6 h were infected with IBDV for an additional 6 and 12 h. Total viral RNA expression and major viral protein (VP1/2/3) expression were analyzed by reverse transcription-quantitative PCR (RT-qPCR) and Western blotting, respectively. (E) Comparison of VP1/2/3 expression in the different lanes in panel D. The densities of VP1/2/3 and β-actin were quantified by ImageJ software. (F) DF-1 cells treated with 5 μg/mL RO3306 or DMSO for 6 h were infected with IBDV for an additional 12, 18, and 24 h. Viral titers of whole cells were examined by 50% tissue culture infective dose (TCID_50_) assay. Western blot data were semi-quantified and normalized against the β-actin protein loading control. Data were obtained from three independent experiments and presented as means ± SD. *, *P < *0.05; **, *P < *0.01; ***, *P < *0.001.

These observations and the key role of CDK1-cyclin B1 kinase activity led us to investigate the role of CDK1-cyclin B1 in viral replication. A well-characterized CDK1 inhibitor, RO3306, was used to treat DF-1 cells (46). First, we examined the effects of RO3306 on cell viability and the kinase activity of CDK1. Pretreatment of cells with 5 μg/mL of RO3306 resulted in no toxic effects on cell viability (Fig. 3A) and increased CDK1Y15 phosphorylation (Fig. 3B). However, CDK1 and cyclin B1 expression were not affected by RO3306 treatment (Fig. 3B). Prior work has shown that increased levels of p-CDK1Y15 indicate suppression of CDK1-cyclin B1 kinase activity (47). Afterwards, we quantified viral replication after treatment with RO3306 or dimethyl sulfoxide (DMSO). It was found that the relative viral genome copy numbers in RO3306-treated cells were significantly decreased by ~70% at 6 h postinfection (hpi) and by ~85% at 12 hpi compared with those in the DMSO-treated cells (Fig. 3C). Consistent with the reverse transcription-quantitative PCR (RT-qPCR) results, the expression of major viral proteins, including VP1, VP2 and VP3, was significantly reduced by ~50% in RO3306-treated cells compared to that in DMSO-treated cells (Fig. 3D, lane 5 versus lane 3; Fig. 3E). Furthermore, we performed a 50% tissue culture infective dose (TCID_50_) assay to examine the effects of RO3306 on IBDV replication. As shown in Fig. 3F, from 12 to 24 hpi, the viral titers were ~50% lower in RO3306-treated cells than in DMSO-treated cells. Thus, these data suggest that inhibition of CDK1-cyclin B1 significantly disrupts IBDV replication.

### Silence of CDK1-cyclin B1 inhibits viral replication.

**FIG 4:**
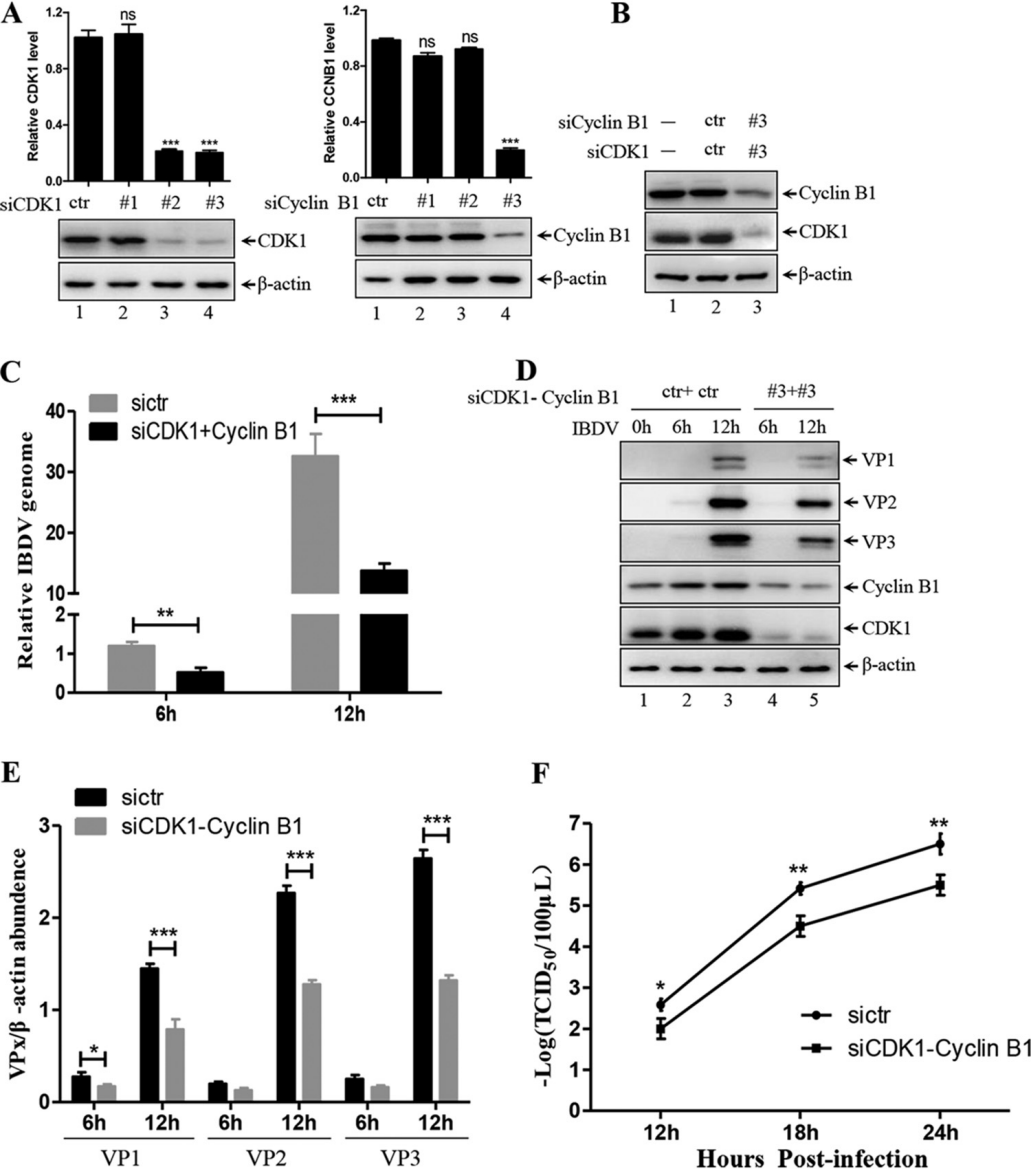
CDK1-cyclin B1 silencing inhibits viral proliferation. (A) Three small interfering RNAs (siRNAs) targeting CDK1, three cyclin B1 siRNAs, and the control siRNA (sictr) were individually transfected into DF-1 cells for 48 h. Whole-cell lysates were subjected to immunoblotting with corresponding antibodies (bottom panel). Comparison of CDK1 and cyclin B1 expression in different lanes. CDK1 and cyclin B1 density were quantified using ImageJ software (top panel). (B) Indicated siRNAs were co-transfected into DF-1 cells for 48 h. The protein levels of CDK1, cyclin B1 and β-actin in transfected cells were examined by Western blotting with indicated antibodies. (C and D) DF-1 cells transfected with indicated siRNAs for 48 h were infected with 1 MOI IBDV for different times. Viral genome (C) and major viral protein VP1/2/3 levels (D) were analyzed by RT-qPCR and Western blotting, respectively. (E) Comparison of VP1/2/3 expression in the different lanes in panel D. VP1/2/3 and β-actin density were quantified using ImageJ software. (F) DF-1 cells transfected with indicated siRNAs for 48 h were infected with 0.1 MOI IBDV for additional different times. Viral titers of whole cells were examined by TCID_50_ assay. Western blot data were semi-quantified and normalized against β-actin protein loading control. Data were obtained from three independent experiments and are presented as means ± SD. *, *P < *0.05; **, *P < *0.01; ***, *P < *0.001; ns, not significant.

The inhibitory effect of RO3306 on IBDV replication led us to investigate whether silencing CDK1-cyclin B1 suppresses viral proliferation in DF-1 cells. To determine this, CDK1-cyclin B1 expression was knocked down in DF-1 cells via small interfering RNAs (siRNAs). First, we designed and screened for efficient siRNA which effectively knocked down CDK1-cyclin B1. The expression of two siRNAs (siRNA 2 and siRNA 3) resulted in an ~80% reduction in endogenous CDK1 expression in DF-1 cells (Fig. 4A, left panel, lanes 3 and 4 versus lane 1). SiRNA 3 targeting cyclin B1 resulted in an ~80% decrease in endogenous cyclin B1 expression in DF-1 cells (Fig. 4A, right panel, lane 4 versus lane 1). Additionally, co-transfection of siCDK1 3 and siCyclin B1 3 led to significantly reduced expression of endogenous CDK1 and cyclin B1 (Fig. 4B, lane 3 versus lane 1). To study the effect of this siRNA-mediated silencing of CDK1-cyclin B1 on IBDV replication, we performed RT-qPCR assays to quantitate relative viral genome copy numbers in siRNA-transfected cells. We found that virus replication was significantly reduced by ~50% at both 6 and 12 hpi compared to that in the control-transfected cells (Fig. 4C). In agreement with the RT-qPCR results, the expression of major viral proteins, including VP1, VP2 and VP3, was noticeably reduced by ~50% at 12 hpi in siRNA 3-transfected cells (Fig. 4D, lane 5 versus lane 3; Fig. 3E). To further investigate the effect of CDK1-cyclin B1 complex silencing on IBDV replication, we performed a TCID_50_ assay. We found that silencing CDK1-cylin B1 complex significantly hampered IBDV replication, with a reduction of 1 log compared to that in the control cells (Fig. 3F). In conclusion, knockdown of CDK1-cyclin B1 complex effectively reduces viral replication, indicating that this complex is crucial for the IBDV life cycle in host cells.

### CDK1-cyclin B1 phosphorylates VP1.

**FIG 5:**
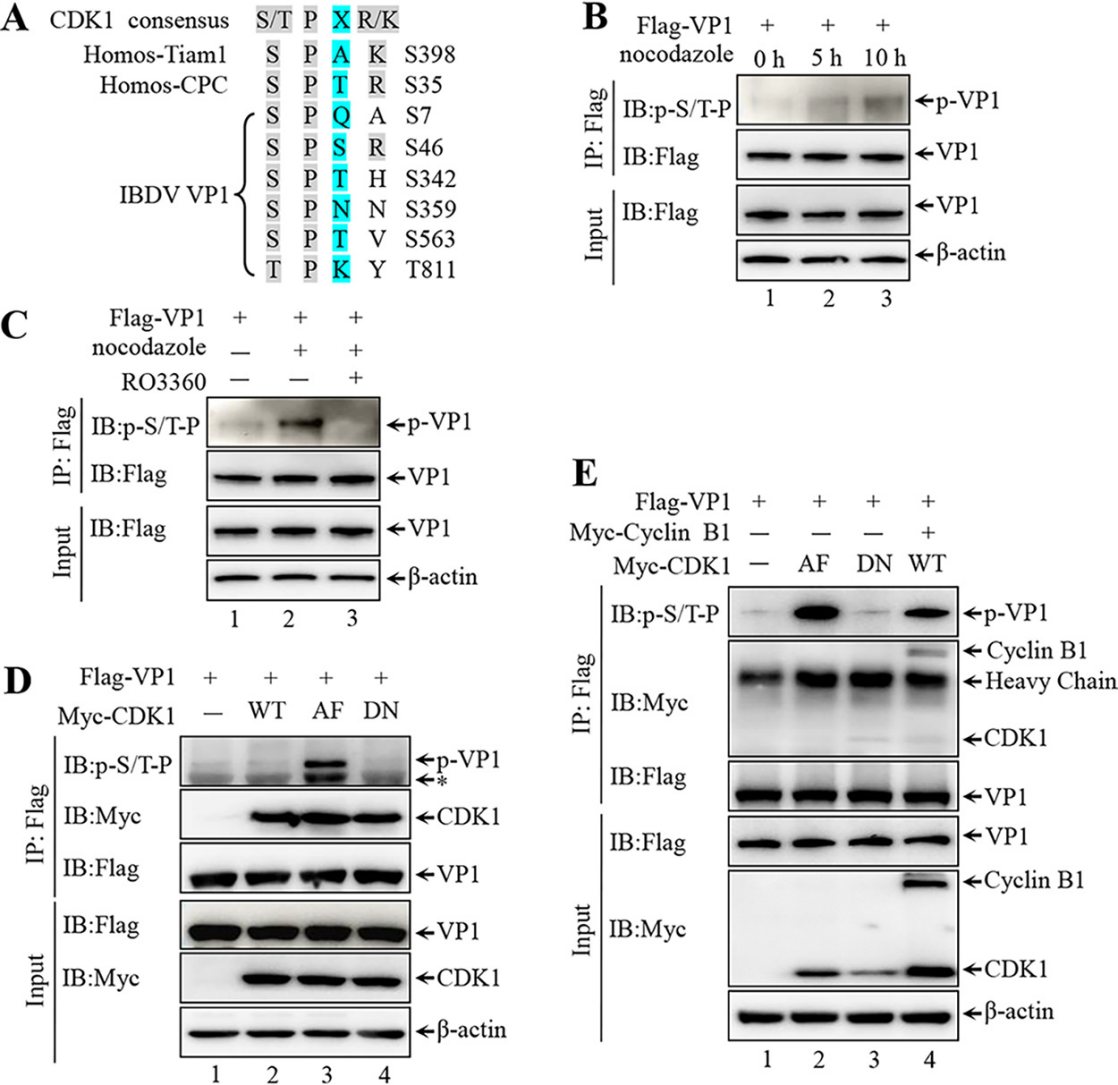
CDK1-cyclin B1 phosphorylates VP1. (A) Schematic diagram of published substrates and putative VP1 phosphorylation sites of CDK1. The multiple S/T-P motifs, positions of S7, S46, S342, S359, S563, and T811, are indicated. (B) 293T cells transfected with Flag-VP1 were treated with 10 nM nocodazole for different durations. Next, the cells were subjected to IP. Immunoblotting of IP complex (Top panel) and the cell lysates (Bottom panel) by Western blotting with indicated antibodies. (C) 293T cells transfected with Flag-VP1 were treated with 10 nM nocodazole or 10 nM nocodazole together with 5 μg/mL RO3306 for an additional 12 h. Next, cells were subjected to IP. Immunoblotting of IP complex (Top panel) and the cell lysates (Bottom panel) by Western blotting with indicated antibodies. (D) The indicated expressing plasmids were co-transfected into 293T cells for 36 h. Transfected cells were subjected to IP. The protein levels of IP complex and cell lysates were analyzed by Western blotting with recommended antibodies. (E) IB of whole-cell lysates (Bottom panel) and proteins immunoprecipitated with anti-Flag agarose (top panel) from 293T cells transfected with the indicated plasmids for 36 h.

The interaction of VP1 and CDK1-cyclin B1 complex prompted us to investigate whether CDK1-cyclin B1 phosphorylates VP1. CDK1 is a proline-orientated kinase which preferentially phosphorylates the consensus motif pS/T-P-X-K/R (p indicates phosphorylation site, X indicates any amino acid), although it can also phosphorylate the minimal sequence pS/T-P (48, 49). It has been reported that several host and viral proteins are phosphorylated by CDK1-cyclin B1 in these two motifs (50, 51). In this study, five minimal CDK1 sequences (S/T-P) and one canonical CDK1 phosphorylation sites (S/T-P-X-K/R) were present in VP1 (Fig. 5A). We used a CDK1 substrate-specific antibody, anti-pS/T-P which recognizes p-S/T-P, and nocodazole, an activator of CDK1, to assess whether CDK1 can phosphorylate VP1. Immunoprecipitation (IP) and immunoblotting of Flag-VP1 from nocodazole-treated 293T cells showed that the level of phosphorylated VP1 increased in a time-dependent manner (Fig. 5B). However, this increased phosphorylation of VP1 was completely reversed by RO3306, a CDK1 inhibitor (Fig. 5C, lane 3 versus lane 2). Consistent with the results of VP1 phosphorylation, immunoblotting the IP complex from 293T cells co-transfected with Flag-VP1 and constitutively active CDK1 (Y14A, Y15F [AF]) or kinase-deficient mutant CDK1 (D145N [DN]) plasmid constructs showed that AF, but not DN, led to strongly increased VP1 phosphorylation (Fig. 5D, lanes 3 and 4) (52). Furthermore, the level of VP1 phosphorylation was noticeably higher with co-transfection of CDK1-cyclin B1 complex than with transfection of CDK1 or DN alone (Fig. 5E, lane 4 versus lanes 1 and 3), but was lower than that with transfection of AF (Fig. 5E, lane 4 versus lane 2). In summary, our results demonstrate that VP1 is a substrate for CDK1-cyclin B1 complex-mediated phosphorylation.

### Serine 7 of VP1 is the phosphorylation site.

**FIG 6:**
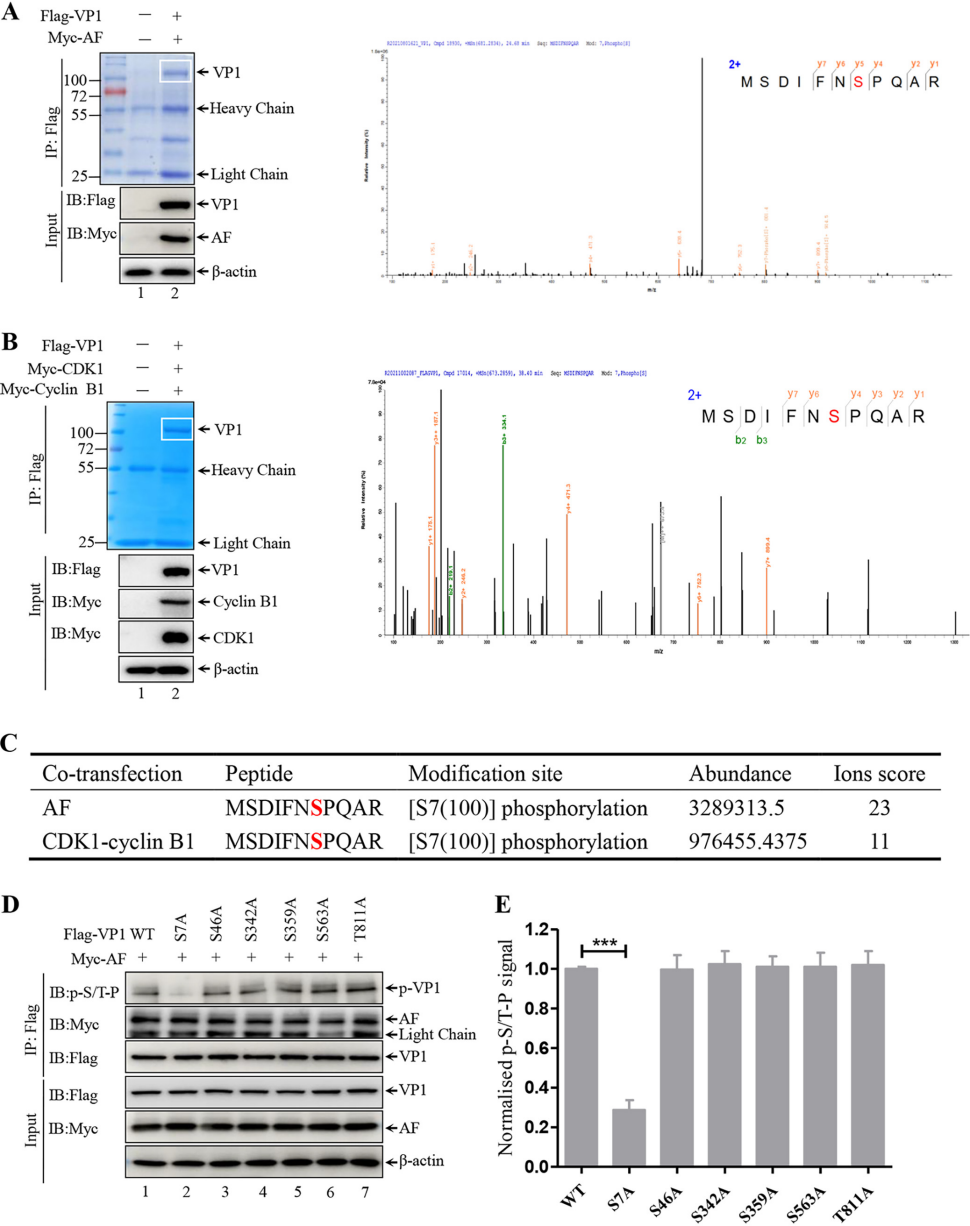
Serine 7 of VP1 is the phosphorylation site. (A) 293T cells were transiently transfected with Flag-VP1- or Myc-CDK1AF-expressing plasmids for 36h. Flag-VP1 was IP with an anti-Flag agarose and separated by SDS-PAGE, and the band corresponding to VP1 (white box) was excised, digested, and analyzed by liquid chromatography-tandem mass spectrometry (LC-MS; left panel). The proteins in cell lysates were also analyzed by Western blotting. The MS spectra of the selected phosphorylated VP1 peptide, MSDIFNSPQAR, is shown in the right panel. (B) Flag-VP1-, Myc-CDK1-, and Myc-cyclin B1-expressing plasmids were co-transfected into 293T cells for 36 h. Flag-VP1 was purified by IP with an anti-Flag agarose and separated by SDS-PAGE, and the band corresponding to VP1 (white box) excised, digested, and analyzed by LC-MS (left panel). The proteins in cell lysates were also detected by Western blotting. The MS spectra of the selected phosphorylated VP1 peptide, MSDIFNSPQAR, is shown in the right panel. (C) Information on the phosphorylated peptide identified by LC-MS, as described in panels A and B. (D) Wild-type (WT) Flag-VP1 and six mutant VP1-expressing plasmids were individually co-transfected with Myc-CDK1AF into 293T cells for 36 h. IB of whole-cell lysates (Bottom panel) and proteins immunoprecipitated with anti-Flag agarose (top) from the transfected cells. (E) Quantitation shows mean p-S/T-P normalized to Flag-VP1 signals shown in panel D. p-S/T-P, Flag-VP1, and β-actin densities were quantified using ImageJ software. Data were obtained from three independent experiments and are presented as means ± SD. ***, *P < *0.001.

As mentioned previously, the consensus region S/T-P-X-R/K and the optimal motif S/T-P are preferentially targeted by CDK1 for phosphorylation (53). VP1 contains six putative phosphorylation sites (Fig. 5A). Mass spectrometry analysis of purified Flag-VP1 from the lysates of 293T cells co-transfected with Flag-VP1 and Myc-AF showed that serine 7 of VP1 may be a phosphorylation site (Fig. 6A and C). To identify the precise site of CDK1-cyclin B1 complex-mediated phosphorylation of VP1, we co-transfected the plasmid Flag-VP1 with Myc-CDK1 and Myc-cyclin B1 into 293T cells. The purified Flag-VP1 band obtained by the IP assay was subjected to mass spectrometry analysis (Fig. 6B, left panel). As shown in Fig. 6B (right panel) and Fig. 6C, serine 7 on the peptide (MSDIFNSPQAR) located in the initial VP1 sequence was a phosphorylation site with high confidence, which is consistent with the results shown in Fig. 6A. To rule out the other five potential phosphorylation sites, Myc-AF was co-transfected with each indicated mutant Flag-VP1 into 293T cells for 36 h. IP and subsequent Western blot analysis showed a significant reduction in the CDK1 phosphorylation signal of S7A compared with that of wild-type (WT) VP1 or the other five VP1 mutants (Fig. 6D, lane 2 versus other lanes; Fig. 6E). Altogether, these data suggest that serine 7 (Ser7) of VP1 is the phosphorylation site mediated by CDK1-cyclin B1.

### Characterization of VP1 S7 phosphorylation antibody.

**FIG 7:**
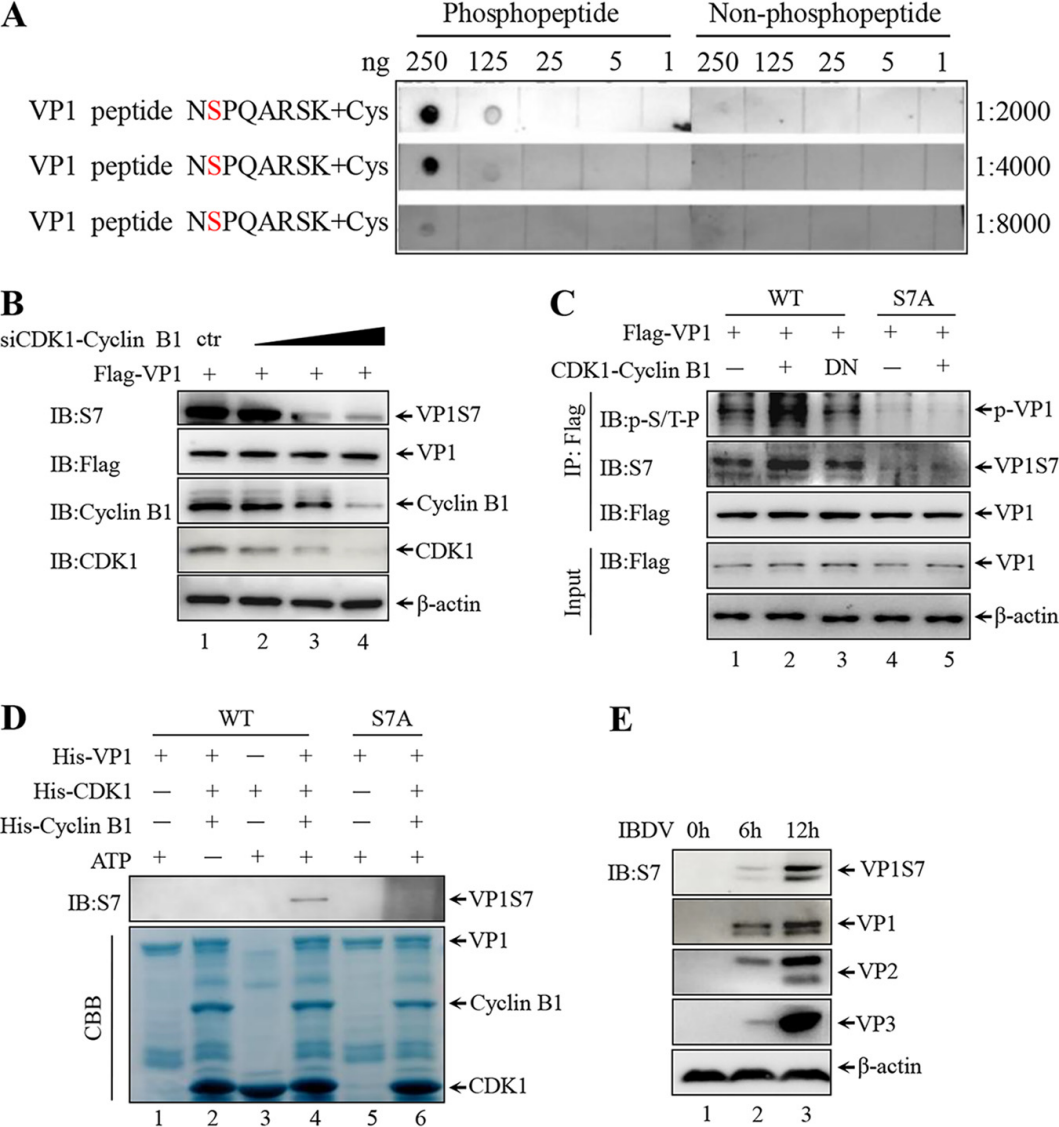
Characterization of specific antibody against VP1 S7 phosphorylation. (A) Synthetic phosphopeptides and non-phosphopeptides were diluted and dotted on nitrocellulose membranes, then analyzed by dot blotting with S7 antibody. Peptide sequences are shown with the serine residue of interest indicated in red. (B) Flag-VP1 was co-transfected with indicated siRNAs into DF-1 cells for 36 h. The transfected cells were subjected to Western blotting with indicated antibodies. (C) 293T cells were transfected with the indicated expressing plasmids for 36 h. Cells were then subjected to IP by anti-Flag agarose. Immunoblotting of IP complex (top panel) and cell lysates (bottom) were performed to analyze indicated protein levels. (D) Purified His-VP1 or S7A proteins were incubated with purified His-CDK1/cyclin B1 kinases for phosphorylation reaction *in vitro*. The reaction complexes were analyzed by Western blotting with the indicated antibodies. The purified proteins were separately by SDS-PAGE and visualized by Coomassie brilliant blue staining. (E) DF-1 cells were infected with 1 MOI IBDV for different durations. Immunoblotting of cell lysates was performed to analyze the indicated proteins.

To investigate whether S7 phosphorylation of VP1 occurs during the viral life cycle, we generated a specific rabbit polyclonal antibody against S7 phosphorylation. A dot blot analysis of the S7 antibody showed that it was highly sensitive in detecting its own designated phosphorylation site in a dose-dependent manner and did not cross-react with non-phosphorylated peptide (Fig. 7A). To further confirm that CDK1-cyclin B1 is the kinase of VP1 S7 phosphorylation, we performed a Western blot analysis of Flag-VP1 transfected and CDK1-cyclin B1-silenced DF-1 cells and demonstrated that knockdown of this complex significantly reduced the level of VP1 S7 phosphorylation (Fig. 7B). Additionally, as shown in Fig. 7C, WT CDK1-cyclin B1, not DN CDK1-cyclin B1, promoted S7 phosphorylation (Fig. 7C, lanes 2 and 3); whereas S7A mutant VP1 was not phosphorylated even in the presence of CDK1-cyclin B1 expression plasmids (Fig. 7C, lanes 4 and 5). This phosphorylation of WT VP1, not S7A mutant VP1, was also detected by p-S/T-P antibody (Fig. 7C). Next, we used S7 antibody to examine whether CDK1-cyclin B1 can phosphorylate VP1 *in vitro*. *In vitro* kinase assays with and without purified His-tagged CDK1-cyclin B1 and purified His-VP1 or His-VP1 S7A confirmed the phosphorylation of VP1 on the serine 7 residue (Fig. 7D, lane 4). The level of S7 phosphorylation increased in a time-dependent manner for up to 12 h after IBDV infection (Fig. 7E), implying that IBDV VP1 is indeed phosphorylated on the serine 7 residue during the viral life cycle.

### Phosphorylation of VP1 contributes its polymerase activity.

**FIG 8:**
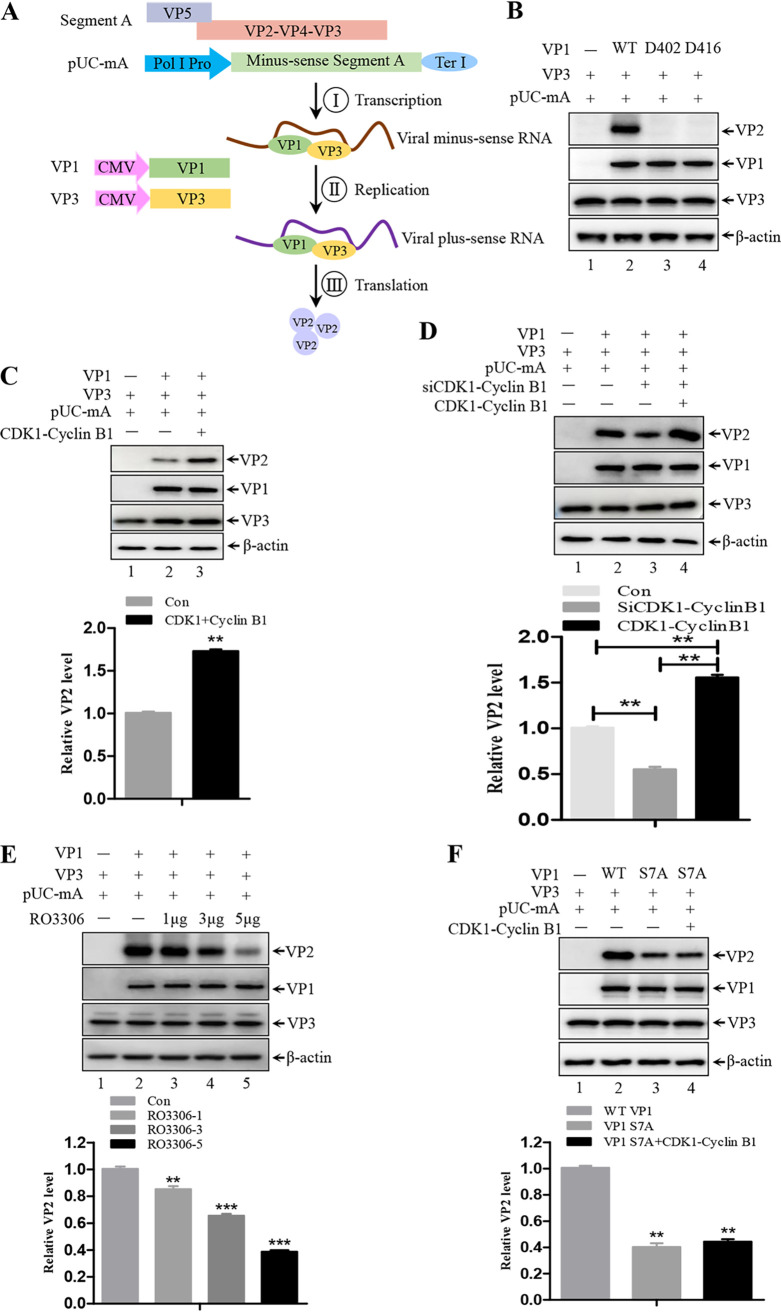
Phosphorylation of VP1 contributes to its polymerase activity. (A) The evaluation system for polymerase activity. (B) VP1- (or D402 and D416 mutant VP1), VP3-, and pUC-mA-expressing plasmids were transfected into 293T cells for 72 h. Cell lysates were subjected Western blotting to analyze VP2, VP1, VP3 and β-actin levels. (C) Top panel: 293T cells were transfected with indicated expressing plasmids for 72 h. Immunoblotting of cell lysates was performed by Western blotting with indicated antibodies. Bottom panel: comparison of VP2 expression in the different lanes shown in the top panel. VP2 and β-actin densities were quantified using ImageJ software. (D) Top panel: DF-1 cells were transfected with the indicated expressing plasmids for 72 h. Immunoblotting of cell lysates was performed by Western blotting with the indicated antibodies. Bottom panel: comparison of VP2 expression in the different lanes shown in the top panel. VP2 and β-actin densities were quantified using ImageJ software. (E) Top panel: DF-1 cells were transfected with the indicated expressing plasmids for 72 h. Transfected cells were treated with different concentrations of RO3306 (1, 3, and 5 μg) for 12 h before being harvested. Immunoblotting of cell lysates was performed by Western blotting with the indicated antibodies. Bottom panel: comparison of VP2 expression in the different lanes shown in the top panel. (F) Top panel: 293T cells were transfected with the indicated plasmids for 72 h. Immunoblotting of cell lysates was performed by Western blotting with the indicated antibodies. Bottom panel: comparison of VP2 expression in the different lanes. VP2 and β-actin densities were quantified using ImageJ software. Western blot data were semi-quantified and normalized against β-actin protein loading control. Data were obtained from three independent experiments and are presented as means ± SD. **, *P < *0.01; ***, *P < *0.001.

According to the X-ray crystallography data, complete VP1 is divided into three critical domains: the N-terminal domain (residues 1 to 168); the central polymerase domain (residues 169 to 657) consisting of the classical “fingers,” “palm,” and “thumb” motif that is crucial for polymerase activity; and the C-terminal domain (residues 658 to 845). The architecture of the initial sequences (residues 1 to 26) of VP1 is disorganized and invisible in the crystal structure (54, 55). The effect of the initial sequences of VP1 on polymerase activity is unclear. Here, by transfecting different combinations of VP1, VP3, and pUC-mA plasmids, we investigated whether S7 phosphorylation affects the VP1 polymerase activity that is necessary for initiating viral genomic RNA translation (56). The pUC-mA plasmid, which generates the minus-sense IBDV segment A transcript after transfection into cells, was used along with VP1- and VP3-expressing plasmids to evaluate polymerase activity by determining the VP2 expression level (Fig. 8A). As shown in Fig. 8B, VP2 was clearly detected in the lysates of 293T cells co-transfected with WT VP1, VP3, and pUC-mA (Fig. 8B, lane 2), but not in the lysates of cells co-transfected with D402A or D416A VP1 or in the absence of VP1 (Fig. 8B, lanes 1, 3, and 4). This is because D402A and D416A VP1 are null mutants of VP1 polymerase, which cannot initiate viral genomic translation. Next, we used this combination system to measure the polymerase activity of VP1. Immunoblotting analyses of different combinations of transfected plasmids showed that ectopic expression of CDK1-cyclin B1 significantly increases VP2 expression compared to the absence of CDK1-cyclin B1 (Fig. 8C, lane 3 versus lane 2). In addition, silencing of CDK1-cyclin B1 causes reduced VP2 expression (by ~50%), which could be restored with expression of CDK1-cyclin B1, compared with the control (Fig. 8D, lanes 3, 4, and 2). In agreement with the inhibitory function of RO3306 in IBDV replication, we next assessed its effect on the polymerase activity of VP1. We found that the CDK1 inhibitor RO3360 significantly reduced VP2 expression in a dose-dependent manner (Fig. 8E). Next, we mutated serine 7 to alanine in VP1 (S7A VP1) to examine the effect of non-phosphorylated VP1 on polymerase activity. As shown in Fig. 8F, the expression of VP2 in 293T cells co-transfected with S7A VP1, VP3, and pUC-mA was significantly lower (~50%) than that in cells co-transfected with WT VP1, VP3, and pUC-mA (Fig. 8F, lane 3 versus lane 2). This reduction in VP2 expression was not restored by the overexpression of CDK1-cylin B1 complex (Fig. 8F, lane 4 versus lane 2). These results demonstrate that the phosphorylation of VP1 on serine 7 mediated by CDK1-cyclin B1 complex is crucial for VP1 polymerase activity.

### Disruption of VP1 phosphorylation damages viral replication.

**FIG 9:**
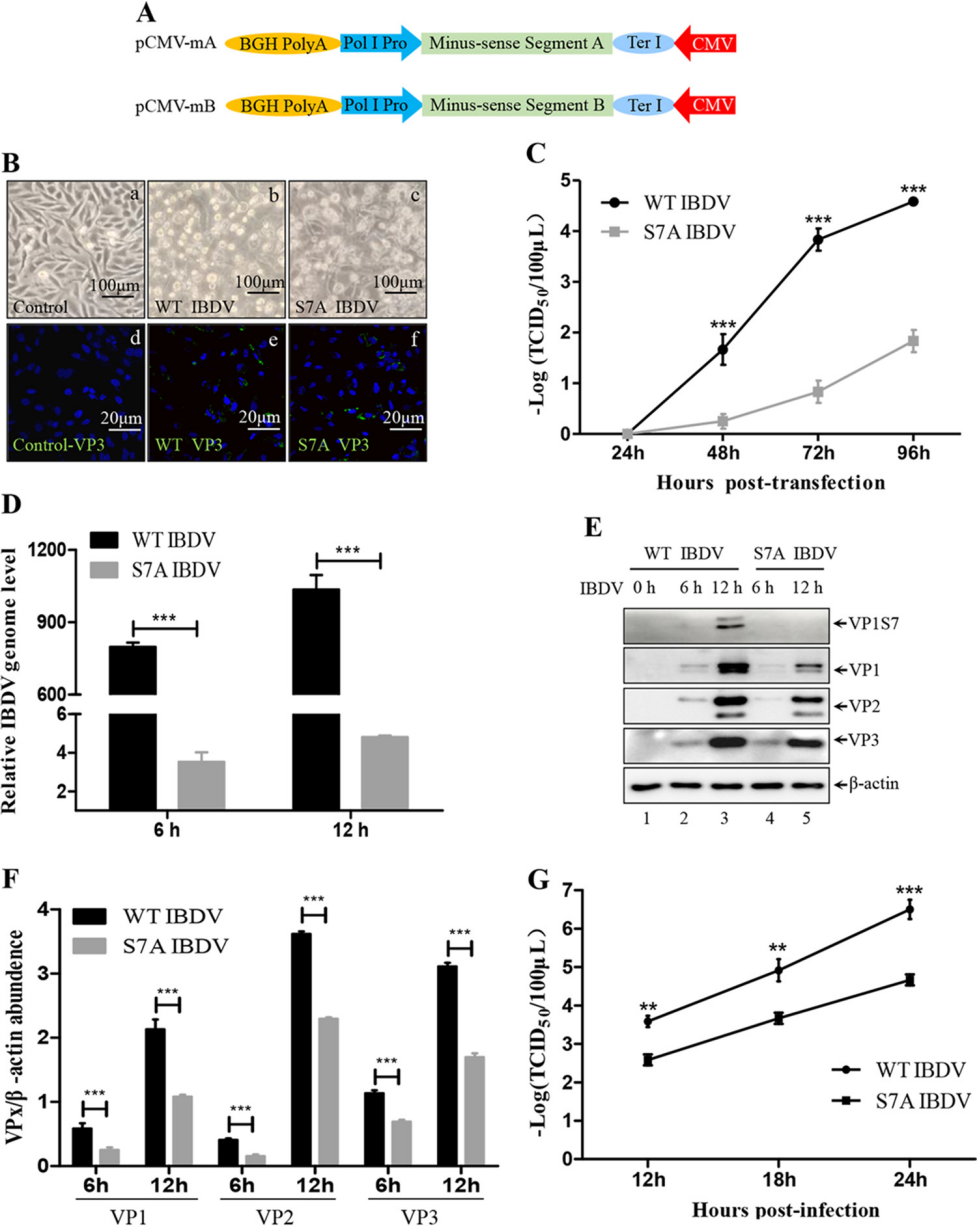
Disrupting phosphorylation of VP1 reduces viral replication. (A) Schematic model of IBDV rescue. This dual-promoter system for viral rescuing was established in a previous report (18). Briefly, Poly I Pro transcripts minus-sense RNA (segment A and B) are in the forward. In the reverse-ward, CMV promoter transcribes the cap-plus-sense RNA (also acting as mRNA) which directly translates viral proteins. Translated proteins subsequently support the replication of minus-sense RNA to produce plus-sense RNA. Viral proteins and complete double-stranded (dsRNA) could be used to produce infectious IBDV. (B) Cytopathic effect images of DF-1 cells infected (or mock-infected) with WT IBDV and S7A mutant IBDV. Scale bars = 100 μm (frames a, b, and c). DF-1 cells infected (or mock-infected) with WT IBDV and S7A mutant IBDV were detected with anti-VP3 (green) antibody. The nuclei were stained by DAPI. Images were scanned by confocal microscopy with 20-μm scale bars (frames d, e, and f). (C) IBDV titers in the supernatants of 293T cells transfected with the indicated plasmids were determined by TCID_50_ assay at 72 and 96 h post-transfection. (D) DF-1 cells were infected with WT IBDV and S7A IBDV (1 MOI each) for different durations. Viral genome levels were determined by RT-qPCR. Results were normalized to GAPDH mRNA levels in the same samples. (E) DF-1 cells were infected with WT IBDV and S7A IBDV (1 MOI each) for different durations. Cell lysates were subjected to Western blotting with anti-VP1, anti-VP2, and anti-VP3 antibodies. β-actin was used as the loading control. (F) Comparison of VP1/2/3 expression in the different lanes in panel E. VP1/2/3 and β-actin densities were quantified using ImageJ software. (G) DF-1 cells were infected with 0.1 MOI WT IBDV or S7A IBDV for different durations (12, 18, and 24 h). Viral titers in supernatants of DF-1 cells were determined by TCID_50_ assay. Western blot data were semi-quantified and normalized against β-actin protein loading control. Data were obtained from three independent experiments and are presented as means ± SD. **, *P < *0.01; ***, *P < *0.001.

In our analysis of 100 complete VP1 sequences, we found that no VP1 sequence containing a serine 7 mutation was available in GenBank. In addition, substitution of the isoleucine 4 (I4) residue of VP1 severely hampers viral replication (57), indicating that Ser7 of VP1 may be critical for IBDV replication. Given that the mutation of serine 7 to alanine could be tolerated while retaining partial polymerase activity, we attempted to rescue an S7A VP1 mutant strain of IBDV. To obtain S7A mutant IBDV, we used a highly effective dual-promoter IBDV rescue system (Fig. 9A) (56). The virus rescue experiment was conducted in 293T cells by co-transfecting pCMV-mA together with pCMV-mBS7A. Co-transfection of pCMV-mA alone and with WT pCMV-mB were included as negative and positive controls, respectively. As shown in Fig. 9B, the supernatants of 293T cells co-transfected with pCMV-mA and pCMV-mB or pCMV-mBS7A could induce visible cytopathic effects (CPE) in DF-1 cells (Fig. 9B, frames b and c). CPE were not observed in DF-1 cells incubated with the supernatants of cells co-transfected with pCMV-mA alone (Fig. 9B, frame a). These rescued viruses were further confirmed by immunostaining with VP3 antibody (Fig. 9B, frames e and f). These results showed that VP1 S7A mutant IBDV was successfully generated. An S7A mutation in VP1 decreases its polymerase activity, which might affect the efficiency of viral rescue. Hence, we compared the effectiveness of WT and S7A VP1 mutant on IBDV rescue. As shown in Fig. 9C, the viral titers of rescued WT IBDV were 3 logs higher than those of the rescued S7A mutant IBDV. These results demonstrate that VP1 S7 is important for polymerase activity, which then affects viral rescue. Next, the growth kinetics of WT IBDV and S7A mutant IBDV were further assessed by RT-qPCR. The results indicated that the relative viral genome copy numbers in S7A IBDV-infected cells were significantly reduced by 200-fold at 6 and 12 hpi compared to those in WT IBDV-infected cells (Fig. 9D). Consistent with the RT-qPCR results, compared to that in WT IBDV-infected cells, the expression of major viral proteins, such as VP1, VP2 and VP3, was noticeably reduced by ~50% at 6 and 12 hpi in S7A IBDV-infected cells (Fig. 9E, lane 4 versus lane 2, lane 5 versus lane 3, Fig. 9F). Moreover, the S7 phosphorylated VP1 was only detected in WT IBDV by an S7-specific antibody, rather than in the S7A mutant IBDV (Fig. 9E). Furthermore, the growth kinetics of WT IBDV and S7A IBDV were further evaluated by titration. As shown in Fig. 9G, the titer of S7A IBDV was noticeably lower than that of WT IBDV, further confirming that a serine 7 mutation causes diminished VP1 polymerase activity, which results in reduced IBDV replication. In conclusion, our data indicate that S7 phosphorylation of VP1 is crucial for viral replication.

## DISCUSSION

Various host cell kinases are hijacked to facilitate virus replication by phosphorylating viral proteins (58, 59). Previously, it has been shown that in many viruses, phosphorylation of polymerase proteins is crucial for their catalytic activity during viral genome replication (14). IBDV VP1 is a polymerase that is known to be post-translationally modified by ubiquitin and SUMO1, both of which support IBDV replication (26, 27). Whether this VP1 is also phosphorylated during infection is still unclear. In this report, we have provided multiple lines of evidence that cellular CDK1-cyclin B1 kinase complex performs phosphorylation of VP1, *in vivo* and *in vitro*, at the serine 7 site which is crucial for viral replication. First, mass spectrometry analysis of purified Flag-VP1 showed that CDK1 interacts with VP1. Co-IP and a GST-pulldown assay verified this interaction *in vivo* and *in vitro*. Second, following IBDV infection, CDK1-cyclin B1 accumulated and co-localized with VP1 at the site of IBDV replication in the cytoplasm. Third, our data showed that CDK1 inhibitor treatment and CDK1-cyclin B1 silencing severely disrupt IBDV replication. Fourth, other evidence, including two mass spectrometry analyses of purified Flag-VP1 and generation of an S7-specific antibody, demonstrate that CDK1-cyclin B1 complex can phosphorylate VP1 at serine 7 *in vivo* and *in vitro*. Finally, quantification of polymerase enzyme activity showed that S7 mutant VP1 had remarkably diminished VP1 activity, which resulted in impaired viral replication.

It has been proposed that CDK1, also known as CDC2, phosphorylates a variety of host and viral proteins in the S/T-P motif, also known as the proline-directed sequence motif (43, 60). Six S/T-P motifs in VP1 prompted us to study the mechanism of CDK1 targeting VP1 for phosphorylation. The p-S/T-P antibody is generally used to detect CDK1-mediated phosphorylation (49). However, there may be slight nonspecific reactions (Fig. 6D, lane 2). Hence, specific VP1 Ser7 antibody was generated to exclude the weak background reaction of p-S/T-P antibody (Fig. 7). CDK1 typically binds cyclin B1 to form a complex that performs its kinase function (61). Our results showed that CDK1-cyclin B1 complex phosphorylates VP1 at serine 7. Although four kinases were confirmed to interact with VP1, the question of whether other kinases target VP1 for phosphorylation on its other residues requires further study. Human cytomegalovirus and human papillomavirus type 16 infection induce G_2_-phase arrest by sustaining active CDK1-cyclin B1 complex in the cytoplasm (45, 62). The severe fever with thrombocytopenia syndrome virus protein NSs interacts with CDK1 to cause G_2_-phase cell cycle arrest, which supports viral replication (63). However, whether IBDV infection affects the cell cycle by hijacking CDK1-cyclin B1 complex is still unclear; our data showed that active CDK1-cyclin B1 accumulated at the site of IBDV replication in the cytoplasm.

Two liquid chromatography-tandem mass spectrometry (LC-MS) analyses together with mutations showed that the Ser7 located in the initial domain of VP1 is the target site of phosphorylation by CDK1. Based on previous reports, the VP1 N-terminal domain (residues 1 to 168) surrounds the central polymerase domain, which is bridged by the fingers and thumb of the catalytic cleft, indicating that the N terminus is critical for VP1 activity (54). Nevertheless, the first 26 residues of VP1 are disordered and not visible in the 3D structure (55), suggesting that the role of these initial sequences of VP1 in modulating polymerase activity is not clear. Previous reports have shown that substitution of the isoleucine 4 residue of VP1 with valine disrupts viral replication (57). Because the Ser7 residue is highly close to the I4, we hypothesize that S7 phosphorylation is essential for polymerase activity and viral genome replication. A 3D structure analysis showed that the N terminus, especially the Ser2 residue, of infectious pancreatic necrosis virus (IPNV) VP1 is essential for the formation of VPg (VP1-dsRNA complex) (64). IPNV is a member of the *Birnaviridae* family (65). Sequence alignment shows that the same initial sequence “MSDIFNSPQ” appears at the N terminus in both IBDV VP1 and IPNV VP1, suggesting that S7 phosphorylation may also occur in IPNV VP1, and that the Ser7 residue of both IBDV and IPNV VP1 is critical for polymerase functions such as genome attachment. To generate Ser7 mutants, we developed an IBDV dual-direct promoter rescue system, which was first utilized in an avian influenza virus reverse genetic system (66). Our data showed that a Ser7 mutation severely disrupted VP1-mediated polymerase activity and viral proliferation. We postulate that IBDV hijacks host kinase CDK1 to phosphorylate VP1 and its activation, which is necessary for IBDV replication. Therefore, this study is the first report showing the crucial role of N-terminal residues of VP1 in its polymerase activity.

Our previous work found that Ubc9, an essential E2-conjugate enzyme for SUMOylation, is recruited to VP1 during IBDV replication; this supports SUMOylation of VP1 stabilizing VP1 via inhibiting its proteasome-mediated degradation (27). In addition, phosphorylation of Ubc9 on Ser71 mediated by CDK1 remarkably promotes the enzyme activity of Ubc9 (67). Whether IBDV infection supports CDK1 phosphorylated Ubc9, further facilitating the SUMOylation of VP1, is unknown, because both S7 phosphorylation and SUMOylation of VP1 facilitate viral replication. The concurrent effects of VP1 SUMOylation and CDK1-mediated Ubc9 phosphorylation on IBDV replication require further investigation. Similarly, the relationships between S7 phosphorylation, SUMOylation, and K63-linked ubiquitination of VP1 are also unknown.

In summary, our research shows that IBDV VP1 is phosphorylated by CDK1-cyclin B1, which supports viral replication. IBDV infection recruits CDK1-cyclin B1 to the VP1 puncta structure, into the host cytoplasm, where it directly phosphorylates VP1. Phosphorylated VP1 significantly increases viral replication. These findings not only increase our understanding of the role of VP1 phosphorylation in IBDV replication, but also help identify novel antiviral targets for suppressing viral multiplication.

## MATERIALS AND METHODS

### Cell culture and virus.

HEK-293T cells obtained from the China Center Type Culture Collection were cultured in Dulbecco’s modified Eagle’s medium (DMEM; Gibco, Carlsbad, CA) supplemented with 10% fetal bovine serum (FBS; FSP500; ExCell Bio, Uruguay). Chicken fibroblast line DF-1 cells (IM-C027) purchased from IMMOCELL (Xiamen, Fujian, China) were maintained in MEM containing 10% FBS (cat no. 16000-044, Gibco, USA). All cell lines were cultured at 37°C with 5% CO_2_. IBDV strain JX, isolated from chicken bursa infected with IBDV, was maintained in our laboratory and adapted to replicate efficiently in both 293T and DF-1 cells.

### Antibodies and reagents.

Anti-Flag mouse monoclonal antibody (H3663) was purchased from Sigma-Aldrich (St. Louis, MO). Mouse anti-Myc (M200212F), anti-β-actin (M20009F), anti-His (M3011), and anti-GST (M20007) monoclonal antibodies and agarose-conjugated anti-Flag (M20012F) were purchased from Abmart (Shanghai, China). Rabbit or mouse anti-VP1, -VP2, and -VP3 polyclonal antibodies were all generated from rabbit or mouse sera by immunization with the respective purified proteins. Rabbit anti-CDK1 (A11420), anti-p-CDK1-Y15 (AP0016), and anti-cyclin B1 (A19037) antibodies were all purchased from ABclonal (Wuhan, China). Anti-p-CDK1-Y15 rabbit monoclonal antibody was purchased from Beyotime (AC207; Shanghai, China). Rabbit anti-cyclin B1 antibody was obtained from Proteintech (28603-1-AP; Nanjing, China). Anti-phospho-Ser/Thr-Pro monoclonal antibody (p-S/T-P) (cat no. 9391) was purchased from Cell Signaling Technology, Inc. (Danvers, MA). RO-3306 (SC6673), nocodazole (S1756), and a subcellular extraction kit (P0027) were purchased from Beyotime (Shanghai, China). Horseradish peroxidase (HRP)-labeled anti-mouse and anti-rabbit IgG were purchased from KPL Industries (Millford, MA). Cell lysis buffer NP-40 (50 mM Tris [pH 7.4], 150 mM NaCl, 1% NP-40) used for Western blotting was purchased from Beyotime (P0013F). Immunofluorescence secondary antibodies, including fluorescein isothiocyanate (FITC)-labeled goat anti-rabbit antibody (A0562) and Alexa Fluor 555-conjugated anti-mouse antibody (A0460) were both purchased from Beyotime. Exfect transfection reagent (T101-01/02) was purchased from Vazyme Biotechnology (Nanjing, China).

### Generation of VP1 pSer7 antibody.

Rabbit polyclonal antibody recognizing phosphorylated VP1 pSer7 (1:1,000) was customized from AtaGenix Biotechnology (Wuhan, China). Peptides containing VP1 pSer7 were injected into rabbits for four rounds. The rabbit serum was collected and purified using an affinity column conjugated with nonmodified peptide to exclude antibodies recognizing non-modified VP1, followed by an affinity column conjugated with VP1 pSer7 peptide to bind to and purify the antibodies. Specific antibodies were then eluted and concentrated.

### Plasmid DNA construction, site-directed mutagenesis, and transfection.

pUC-mA, pCMV-mA, pCMV-mB, Flag-VP1, pCDNA3.0-VP1, and pCDNA3.0-VP3 were generated according to previous methods and stored in our laboratory (19). Site-directed mutagenesis was performed as previously described to produce expression vectors for Ser (S) to Ala (A) mutation (68). Myc-tagged chicken CDK1- and cyclin B1-expressing plasmids were all constructed by Tranship Biotechnology (Shanghai, China). Constitutively active CDK1 (T14A, Y15F [AF]), kinase-dead mutant (D145N [DN]), and VP1 mutant plasmids were generated by site-directed mutagenesis (51). All recombinant plasmids were confirmed via Sanger sequencing. All plasmid DNA was transfected into cells using Exfect transfection reagent based on previous protocols.

### Reverse transcription and RT-qPCR.

Six-well plates of DF-1 cells were infected with IBDV at different times. The total RNA was extracted from the cells at 0, 6, and 12 hpi using TRIzol reagent (Beyotime) based on the manufacturer’s instructions. One μg RNA was subjected to reverse transcription using SynScript III cDNA Synthesis Mix (TSK322S; Tsingke Biotech, Beijing, China). Amplification of the target genes (*cdk1*, *cyclin b1*, viral genome, and *gapdh*) was performed to analyze transcript abundance; the transcription level of *gapdh* was determined as the internal control. The primers used are listed in Table 1. RT-qPCR was performed using 2× Tsingke Master qPCR Mix (SYBR Green I) (TSE20; Tsingke Biotech) with a QuantStudio 7 Flex Real-time PCR Detection System (ABI7900, Applied Biosystems, Waltham, MA). The primer sequences for RT-qPCR are listed in Table 1. Gene transcription levels were determined by threshold cycle (2^–ΔΔ^*^CT^*) calculation.

**TABLE 1:** Primers used in this study^*a*^

Primer	Sequence
chCDK1	
F	GTGAGGAGGAAGGTGTTCCA
R	AACACGCGAACGATCCAAAT
chCCNB1	
F	AGGATCCAATGTGCCCAAGA
R	TCACAGCAGCTGGAGGTTTA
siControl	GCATGTCGATGCAGCTACGC
siCDK1	
1	AAGGTGTTCCAAGTACTGCTA
2	AAGTACTGCTATCCGAGAAAT
3	AAGACGAGTTCTGCACAGAGA
siCCNB1	
1	AAGACATCGATGCGGATGACT
2	AAGATGACAAACACGGTTGTA
3	AAGAGATGTACTCTCCTGATA
IBDV	
F	CCTCTGGGAGTCACGAATTAAC
R	ACTCATGGTGGCAGAATCATC
chGAPDH	
F	CCCAGCAACATGAAATGGGCAGAT
R	TGATAACACGCTTAGCACC

a CDK1, cyclin-dependent kinase; IBDV, infectious bursal disease virus.

### Indirect immunofluorescence assay and confocal scanning microscopy.

DF-1 cells cultured in a confocal dish (Nest, China) were transfected with Myc-CDK1 or Myc-cyclin B1 plasmids for 24 h and then infected with IBDV (MOI = 1) for an additional 12 h. The cells were then washed twice with cold phosphate-buffered saline (PBS), fixed with 4% paraformaldehyde for 10 min, and penetrated with 0.2% Triton X-100 for 5 min at room temperature. After extensive washing five times with PBS, the fixed cells were subsequently incubated with mouse anti-VP1 antibody and rabbit anti-Myc antibody for 2 h at room temperature. After four washes with cold PBS, the cells were stained with Alex Fluor-555-conjugated goat anti-mouse IgG and FITC-labeled goat anti-rabbit IgG for 1 h at 37°C. Finally, the nuclei were stained with DAPI (4′,6-diamidino-2-phenylindole). Fluorescence signals were scanned using an Olympus laser scanning confocal microscope (Olympus Corporation, Tokyo, Japan).

### Co-IP assay.

293T cells seeded in 6-well plates were transiently co-transfected with the indicated plasmids for 36 h. The cells were washed three times with cold PBS and lysed in NP-40 buffer containing phosphatase inhibitor cocktail A (P1081; Beyotime) for 30 min at 4°C. After centrifugation of the cell lysates at 12,000 × *g*, the supernatants were incubated with 10 μL anti-Flag beads for 4 h at 4°C. Next, the beads were collected by centrifugation at 2,000 × *g* for 3 min at 4°C and washed four times with cold NP-40 buffer. The final sample proteins were eluted by boiling in SDS-PAGE sample loading buffer (P0015L; Beyotime). Finally, the protein samples were subjected to SDS-PAGE and Western blotting with the recommended antibodies.

### Western blot analysis.

Western blot analysis was performed as described previously (69). Briefly, protein samples were prepared under different conditions. Whole-cell lysates were extracted by NP-40 buffer at 4°C for 15 min, and centrifuged at 12,000 × *g* at 4°C for 10 min. The supernatants were eluted in 4× SDS loading buffer. Equivalent volumes of samples were separated on SDS-PAGE and transferred to nitrocellulose membranes (GE Healthcare, Chicago, IL). The membranes were blocked with 5% skim milk for 30 min at room temperature, then incubated with the indicated primary antibodies at 4°C for 12 h. Subsequently, after three washes with PBS (5 min each time), the membranes was incubated with HRP-conjugated secondary antibody at room temperature for 1 h. Finally, after incubation with enhance chemiluminescence (ECL) reagent, the signals of the membranes were scanned using an AMERSH Amersham ImageQuant 800 (AI800) (GE Healthcare). The histograms of each Western blot analysis were plotted by semi-quantification assay according to previous methods (70). The expression levels of each protein were presented as the ratio of protein/β-actin according to the densities of bands scanned by ImageJ software from three independent assays.

### GST-pulldown.

For the GST-pulldown assay, His-CDK1, His-cyclin B1, and GST-VP1 proteins were individually expressed in *E. coli* BL21 component cells. Both His-CDK1 and His-cyclin B1 were subsequently purified using Ni-NTA agarose. GST-VP1 was purified using a Pierce Glutathione Agarose kit (P2262, Beyotime). Next, 100 ng of GST-VP1 protein was individually added to incubate with the His-CDK1 and His-cyclin B1 proteins for 4 h at 4°C. Next, a 100-μL volume of glutathione agarose beads was added to capture the bait proteins. After three washes with cold PBS (5 min each), the beads were boiled with 4× SDS loading buffer. Finally, the protein samples were subjected to SDS-PAGE and immunoblotted with anti-GST and anti-His antibodies.

### Kinase assays *in vitro*.

For *in vitro* kinase assays, His-tagged chicken kinases (His-CDK1 and His-cyclin B1) were purified from *E. coli* BL21. Purified His-VP1 (~100 ng) was incubated with ~100 ng of kinases in kinase assay buffer (70 mM HEPES, 1 mM MgCl_2_, 3 mM MnCl_2_, 3 μM Na-orthovanadate, 1.2 mM dithiothreitol, 50 μg/mL PEG 2000, and 2 mM ATP) with shaking at 30°C for 1 h. The reaction was stopped by the addition of 4× SDS loading buffer. Finally, the phosphorylated protein was analyzed by Western blotting with the recommended antibodies.

### RNA interference.

To knock down chicken CDK1 and cyclin B1, three siRNA sequences targeting different regions of chicken CDK1 and cyclin B1 mRNA transcripts were designed and synthesized by Tranship Biotechnology (Shanghai, China). In addition, one control siRNA sequence (siCtr) was also designed (Table 1). Six million DF-1 cells were transfected with the indicated siRNAs. The efficiency of RNA silencing was examined by Western blotting at 36 h post-transfection.

### Cellular fractionation.

A nuclear and cytoplasmic protein kit (P0027; Beyotime) was used to isolate nuclear and cytoplasmic components as previously described (68). Briefly, mock- or IBDV-infected DF-1 cells were treated with 200 μL buffer A containing protease inhibitor on ice for 10 min. Next, buffer B was added to a vigorous vortex mix with buffer A, which was centrifuged at 12,000 × *g* for an additional 10 min. The nuclear debris pellet was re-suspended in 100 μL nuclear protein extraction buffer for 20 min. Western blotting was conducted on the supernatant with anti-VP1, anti-S7, anti-CDK1, anti-cyclin B1, and anti-CDK1Y15 antibodies. Anti-H3 and anti-β-actin bands were determined as nuclear and cytoplasmic markers, respectively.

### Mass spectrometry analysis.

293T cells cultured in 60-mm dishes were transfected with the indicated plasmids for 36 h. Whole cells were lysed in NP-40 buffer containing phosphatase inhibitors and protease inhibitor. Cell lysates were incubated with 40 μL anti-Flag beads at 4°C for 2 h. Next, the beads were collected by centrifugation at 2,000 × *g* for 3 min at 4°C and washed four times with cold NP-40 buffer. The final sample proteins were eluted by boiling in SDS-PAGE sample loading buffer and separated by SDS-PAGE. Protein bands visualized via Coomassie brilliant blue staining, excised from SDS-PAGE gel, and digested in 50 mM ammonium bicarbonate buffer containing 200 ng modified sequencing grade trypsin overnight at 37°C. The digested samples were determined using high-sensitivity LC-MS with a QE mass spectrometer at Applied Protein Technology (Shanghai, China).

### Reverse genetics.

The mutant IBDV was rescued using a dual-promoter system (Fig. 8A) as described previously (56). The viral rescue plasmids, pCMV-mA and pCMV-mB, were constructed and preserved in our laboratory. The mutant plasmid pCMV-mBS7A was constructed using a direct mutation assay. The pCMV-mB and pCMV-mB S7A plasmids were co-transfected with pCMV-mA into 293T cells, respectively. At 72 h post-transfection, the resulting cells were freeze-thawed three times, followed by centrifugation at 12,000 × *g* for 10 min. The supernatants were transferred into fresh DF-1 cells and cultured for an additional 48 h until the cytopathic effects were obvious. An immunofluorescence assay with VP3 antibody was used to confirm whether IBDV was rescued successfully.

### TCID_50_ assay.

The viral titers in cell cultures were titrated using a TCID_50_ assay as described previously (27). Briefly, the viral solution was serially diluted 10-fold in MEM containing 2% FBS. A 100-μL aliquot of each diluted sample was added to infect fresh DF-1 cells in 96-well plates. Tissue culture wells with cytopathic effects were considered positive.

### Statistical analysis.

All data were presented as mean ± standard deviation for each group and analyzed using GraphPad Prism version 5.0. The statistical significance of differences between groups was determined using a Student’s *t* test. *P* values less than 0.05 were recorded as statistically significant.
